# Acute Thoracic Aortic Dissection (Stanford Type B) Complicated with Acute Renal Failure

**DOI:** 10.1155/2013/693435

**Published:** 2013-11-12

**Authors:** Li Li, ShunJiu Zhuang, ShaoHong Qi, JiaSheng Cui, JunWen Zhou, Huaqi Zhu, Wan Zhang, Ming Li, Weiguo Fu

**Affiliations:** ^1^Department of Vascular Surgery, Shanghai Huadong Hospital Affiliated with Fudan University, Shanghai 200040, China; ^2^Department of Radiology, Shanghai Huadong Hospital Affiliated with Fudan University, Shanghai 200040, China; ^3^Department of Vascular Surgery, Shanghai Zhongshan Hospital Affiliated with Fudan University, Shanghai 200032, China

## Abstract

We report a recent case and review some literatures of acute aortic dissection (AAD) Stanford type B complicated with late onset of acute renal failure. The patient underwent preoperational peritoneal dialysis followed by thoracic endovascular aortic repair (TEVAR) and was fully recovered and discharged soon after surgery. We conclude that an AAD case is difficult to achieve a timely diagnosis, but with attention to systemic symptoms and dedication thorough treatment plan, a full recovery and positive prognosis are expected.

## 1. Case Report

**Figure 1:**
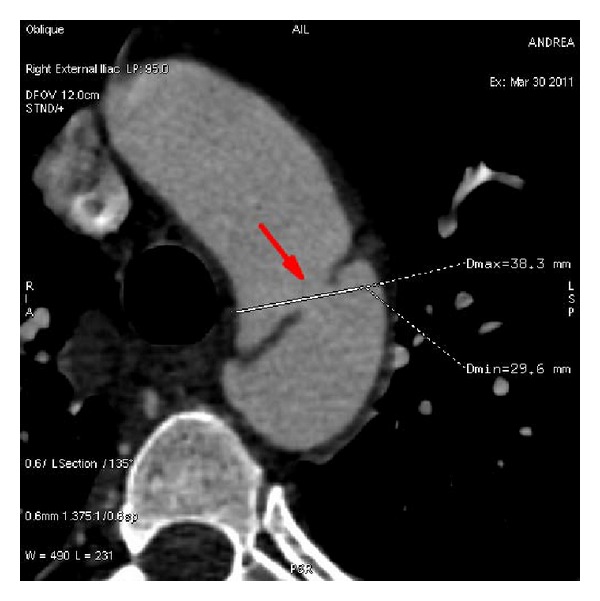
CTA image shows the location of tear in thoracic aorta (arrow).

A 51-year-old male patient was transferred to our unit with a diagnosis of acute thoracic aortic dissection. He had experienced sudden onset excruciating chest-back “tearing” pain with nonspecific ST change for 3 hours after myocardial infarction was ruled out by the cardiac care unit. Upon admission, physical examination showed BP 200/110 mmHg with HR 92; femoral artery was palpable bilaterally. Patient did not have a medical history of hypertension or diabetes and lab testing showed nothing remarkable. As per computed tomography angiography (CTA), the initial rupture in thoracic aorta was located 2 mm distal from the left subclavian artery (LSA), (Figure 1) with dissection extending down to the branch of abdominal aorta and partial involvement of left iliac artery; bilateral renal arteries were both opened to true lumen; celiac trunk opened partially to true lumen; inferior mesenteric artery opened to false lumen; the inner diameter of aortic arch was 31.9 mm. Thus, Stanford type B aortic dissection was confirmed. 

**Figure 2:**
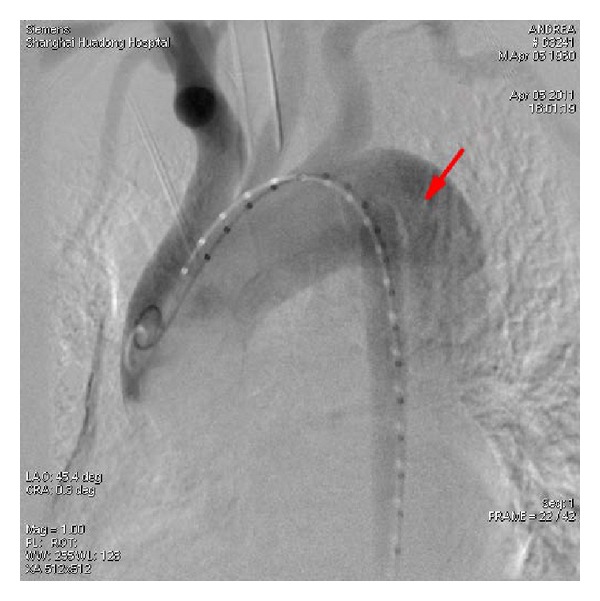
DSA image reveals both true and false lumens (arrow).

**Figure 3:**
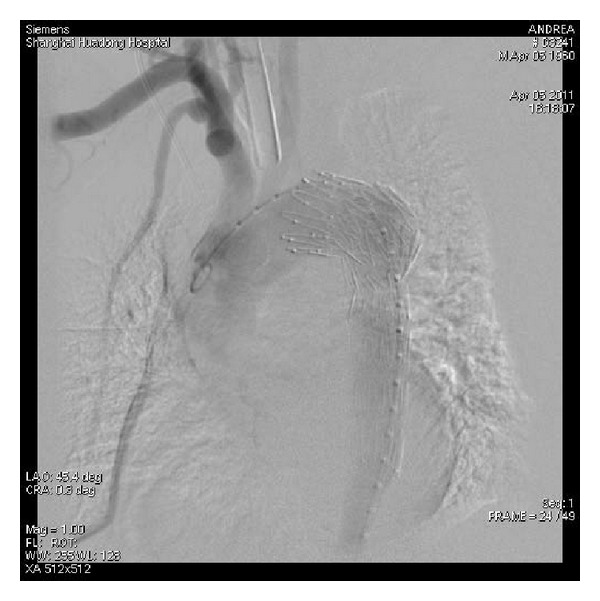
False lumen disappeared under DSA right after graft-stent was inserted into place.

Initial medical management was symptomatic, focusing on controlling blood pressure and heart rate, sedation, and pain relief along with a frequent monitoring schedule to evaluate patient's hemodynamic change, peripheral vascular change, and mental status. Patient was stabilized soon with blood pressure down to 120/75 mmHg. However, four days later, the patient started experiencing increasing chest-back pain, accompanied by ongoing oliguria (from 900 mL/24 hr to 350 mL/24 hr), anuria, facial edema, and agitation; lab testing found that BUN went up to 30.1 mmol/L and creatinine to 710 nmol/L. Acute ischemic renal failure was given the nature of urgency, the peritoneal dialysis was performed immediately. Within 24 hrs, patient's symptoms and hemochemistry were stabilized, and TEVAR was performed under epidural anaesthesia. A pigtail catheter was inserted to the beginning of the ascending aorta via right femoral artery along the lumen over the guide wire for imaging. The digital subtraction angiography (DSA) revealed intact bilateral vertebral artery without left vertebral artery dominance and no impairment above the aortic arch; the rupture was close to the lateral side of the distal opening of the LSA (Figure 2); the aortic lumen was extremely narrowed with very low blood flow; the bilateral renal artery barely showed up due to widely extended false lumen. We decided to proceed with a 38 × 150 mm Talent thoracic stent-graft system (Medtronic, Minneapolis, MN, USA) and cover the LSA ostium in order to obtain enough landing zone. Angiogram confirmed a precise placement of the stent-graft; thoracic aorta rupture was closed completely without any leak; true lumen was extended and false lumen was closed (Figure 3). The bilateral renal arteries, celiac trunk, and superior mesenteric artery were also revealed completely. 

**Figure 4:**
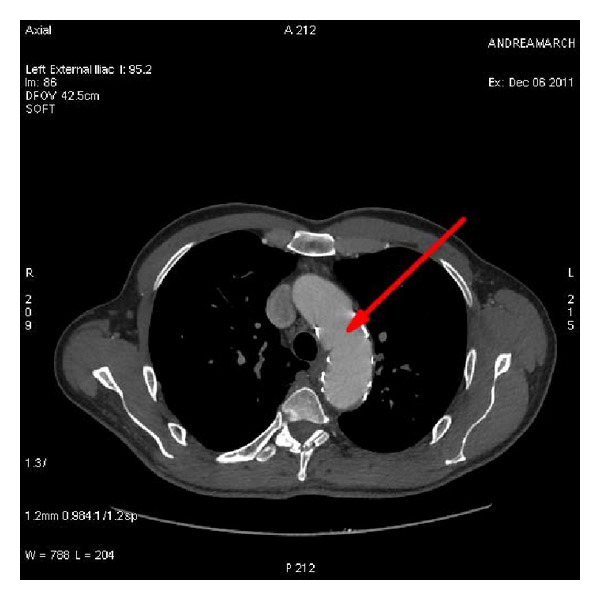
Six-month follow-up CTA shows the thoracic aorta without false lumen.

**Figure 5:**
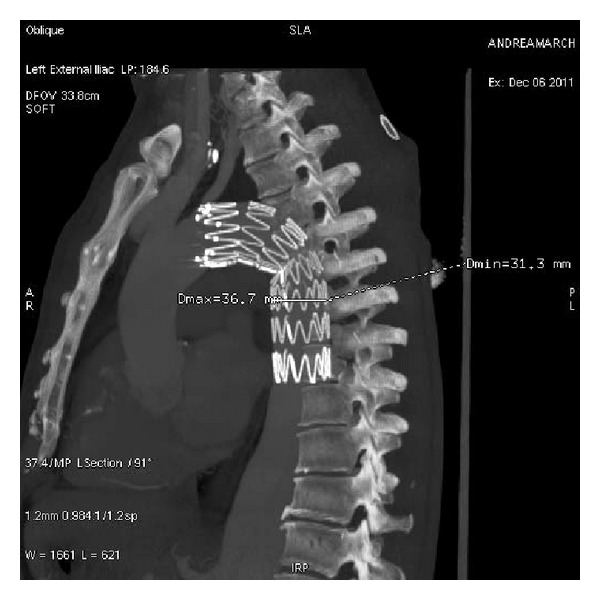
Six-month follow-up CTA reveals that stent-graft placement was intact.

The urine volume was 800 mL 5 hours after surgery and reached 2800 mL after 24 hours. Blood potassium was 4.2 nmol/L. BUN and creatinine levels all went back to normal limits. The patient experienced a full recovery and was discharged 7 days after surgery. Six-month follow-up showed that false lumen disappeared completely and stent-graft placement was intact (Figures 4 and 5).

## 2. Discussion and Literature Review

Aortic dissection occurs when a tear of intima in the aorta leads to the leak of high blood pressure into the media, causing further separation of the two layers downstream. As a result, the true lumen may be pressed or even blocked by the surrounding false lumen, which may cause serious consequences such as internal bleeding, renal failure, intestinal ischemia/necrosis, limb ischemia, and even death. Risk factors include age, hypertension, diabetes, and atherosclerosis [1–3]. While this disease is uncommon, it has high in-hospital mortality rate, due to the fast progress nature and the difficulty of formulating an accurate diagnosis timely.

The International Registry of Acute Aortic Dissection (IRAAD) reviewed 464 patients and found that AAD occurs at a rate of 5 cases per million people in a year, mainly in males with age >60, and 2/3 of them were Stanford type A [3]. While acute symptoms are diverse, sudden onset of chest pain occupies a majority of them; many times it is the only one [3]. Hence, AAD is very often considered acute coronary syndrome (ACS) at the beginning stages of the disease. In our case, the patient showed excruciating chest-back pain for the first few hours with EKG change and was first seen by the cardiac care unit to rule out myocardial infarction. This is usually when an accurate diagnosis and proper medical management are delayed. Although chest pain is entirely a subjective expression, Ramanath et al. suggested that the detail description of the pain sometimes provides clinicians a clue for further investigation. They found that AAD patients tend to describe chest pain as “sharp” “tearing” or “ripping,” and the pain of ACS usually begins gradually and is less severe. Furthermore, anterior chest pain is often associated with type A AAD while type B sometimes shows back or abdominal pain [4]. We found that this is true in this case.

Since 1999, when Dake et al. [5] first applied endovascular stent-graft to repair acute thoracic aortic dissection (TEVAR) successfully, it has rapidly replaced open surgery due to its lower risk and relative simplicity. To this date, a couple of literatures has shown that survival rate of 3–5 years after surgery is fairly high [6, 7]. However, it is commonly agreed that initial medical management should be considered if no evidence of complication is present. Thus, a close monitoring schedule and the ability of accurate assessment of possible complication are critically important. 

We contribute the successful treatment of this case to the following. Firstly, the patient was timely diagnosed and treated. This patient did not show a typical profile of ADD with fairly old age (>60) and a history of hypertension and/or diabetes. He was initially admitted to the cardiac unit to rule out myocardial infarction due to his chest pain and abnormal EKG. However, a CTA test was quickly ordered and an accurate diagnosis was established within a few hour of the onset. Initial medical treatment regime was able to effectively control the blood pressure meanwhile putting patient under a close monitoring schedule for potential complications. Hence, surgical repair was able to be performed with a timely matter when patient started showing renal failure symptom at the early reversible stage.

Secondly, we did not rush into surgery after sign of acute renal failure appeared. Instead, patient underwent an emergent dialysis to control the fluid overload and high blood potassium. The choice of dialysis was peritoneal dialysis due to the simplicity, the nature of urgency, and the upcoming surgery. We found that this thorough pre-op preparation was critical for the fast recovery after surgery in this case.

Lastly, the close proximity between LSA ostium and the tear in this case was put into special concern. An ideal landing zone is key to the success of the surgery to prevent the stent-graft from moving and endoleak [8]. It was recommended that a 15 mm landing zone is optimal for fixation of the stent-graft without covering the ostium of LSA in order to preserve the antegrade flow of LSA [9, 10]. However, with only a 2 mm distance and the acute nature of the disease progress in this patient, we decided to perform a vertebral artery angiography during the surgery and found no presence of a dominant left vertebral artery, which allowed us to perform the repair by covering LSA without concomitant LSA revascularization. Postsurgery exam showed neither sign of neurological dysfunction caused by ischemia or infarction nor hand and upper limb ischemia. 

## 3. Conclusion

Acute aortic dissection has a low incidence however with high in-hospital mortality. “Typical” patients are males with age between 60 and 72 with a history of hypertension or diabetes. The high mortality rate is due to delayed diagnosis and the emergent nature of the disease progress. In addition to timely diagnosis, a successful treatment regime consists of several components including comprehensive medical management plan incorporated with close monitoring schedule, precise assessment of potential complication, (if indicated) thorough pre-op preparation, and careful surgical plan.
